# Fantastic beasts and how to study them: rethinking experimental animal behavior

**DOI:** 10.1242/jeb.247003

**Published:** 2024-02-19

**Authors:** Siyu Serena Ding, Jessica L. Fox, Andrew Gordus, Abhilasha Joshi, James C. Liao, Monika Scholz

**Affiliations:** ^1^ Max Planck Institute of Animal Behavior, 78464 Konstanz, Germany; ^2^ Centre for the Advanced Study of Collective Behaviour, University of Konstanz, 78464 Konstanz, Germany; ^3^ Department of Biology, Case Western Reserve University, Cleveland, OH 44106, USA; ^4^ Department of Biology, Johns Hopkins University, Baltimore, MD 21218, USA; ^5^ Departments of Physiology and Psychiatry, University of California, San Francisco, CA 94158, USA; ^6^ Department of Biology, The Whitney Laboratory for Marine Bioscience, University of Florida, St. Augustine, FL 32080, USA; ^7^ Max Planck Research Group Neural Information Flow, Max Planck Institute for Neurobiology of Behavior – caesar, 53175 Bonn, Germany

**Keywords:** Behavior, Natural habitat, Neuroethology, Non-model organisms

## Abstract

Humans have been trying to understand animal behavior at least since recorded history. Recent rapid development of new technologies has allowed us to make significant progress in understanding the physiological and molecular mechanisms underlying behavior, a key goal of neuroethology. However, there is a tradeoff when studying animal behavior and its underlying biological mechanisms: common behavior protocols in the laboratory are designed to be replicable and controlled, but they often fail to encompass the variability and breadth of natural behavior. This Commentary proposes a framework of 10 key questions that aim to guide researchers in incorporating a rich natural context into their experimental design or in choosing a new animal study system. The 10 questions cover overarching experimental considerations that can provide a template for interspecies comparisons, enable us to develop studies in new model organisms and unlock new experiments in our quest to understand behavior.

## Teaser

**Summary:** Novel technologies enable behavioral experiments with non-model species, in naturalistic habitats and with underexplored behaviors. Consequently, we require a deeper understanding of the natural ecology and lifestyle of study animals.

## Introduction

In 2015, the *New York Times* published a column titled ‘To Fall In Love With Anyone, Do This’ (Catron, 2015). The column described a set of 36 questions to ask a prospective or current partner, intending to foster intimate conversation and a sense of attachment. It also recommended staring directly into each other's eyes for exactly 4 minutes. The *New York Times* promised that with gentle inquiry and extended observation, one could fall in love with anyone. As neuroethologists, many of us entered the field because of our deep affection for animals and the interesting behaviors that they perform in their natural habitats. But to find a good partner animal for our experiments, we needed to be pragmatic. As we progressed in our studies, some of us chose to study more established model systems to better answer mechanistic questions. Our questions, by necessity, became narrower, and were asked of a smaller subset of animals, in a laboratory environment.

**Glossary**

**C-start**

A fast, 5–10 ms latency startle response driven by a network of reticulospinal neurons that simultaneously contracts axial muscle on the side opposite a threatening stimulus, bending the body into a characteristic ‘C’ shape (in fish).

**Central complex**

An arthropod brain region that, in insects, is important for adaptive sensorimotor processing and navigation.

**Gain modulation**

Altering the sensitivity of a sensory system through various processes that can lead to selective amplification or suppression of certain inputs.

**Introgression**

Repeated crossing of an interspecies hybrid with one of the parent species, typically used experimentally to obtain animals with variable genetic material from the parent species.

**Pithing**

Mechanical damage to the brain by a rod or wire.

**Pleiotropic traits**

Two or more seemingly unrelated traits influenced by the same gene.

**Quantitative trait locus (QTL)**

A region of the genome responsible for a specific phenotype.

**Single-cell atlas**

A cell-type reference defined by groups of cells that have similar DNA transcription profiles.

**Social reward**

Positive reinforcement from interaction with conspecifics.

Each of the authors of this Commentary has studied a model system and model behaviors for many good reasons: the application of powerful technologies, the ease of experimentation and husbandry, and the support of an established intellectual community. We came together with the acknowledgment that incentive structures in science can limit the time available to collect data; hence stereotyped behaviors that are easily reproduced in the lab, such as escape behaviors, often enjoy disproportionate representation in the literature. However, many natural behaviors, such as hibernation or migration, require rare and/or highly specific environments (Geiser, 2013; Kubelka et al., 2022). If we are not aware of this trend, the literature will continue to be biased by studies of animal generalists performing a small subset of easily induced behaviors (Cook et al., 2022). An additional challenge of studying rare or variable behaviors arises during data analysis, which may require novel behavioral and pose analysis tools (Wiltschko et al., 2015; Mathis et al., 2018; Pereira et al., 2019; Walter and Couzin, 2021), appropriate statistics and new computational models (Egnor and Branson, 2016), and capabilities for handling very large datasets (von Ziegler et al., 2021). We believe that those of us studying classic model systems could greatly benefit from incorporating the natural contexts and comparative approaches found in other areas of behavioral research such as neuroethology, collective behavior and conservation biology.

Over the last decade, technical advances have propelled us beyond the classical laboratory paradigms and have enabled us to quantitatively investigate relevant behaviors in natural habitats (Kano et al., 2018; Dennis et al., 2021; Guisnet et al., 2021; Koger et al., 2023). New tools beget new opportunities: many organisms can now be studied directly in the field using quantitative approaches that were previously impossible, and the advent of gene-editing technologies is allowing us to manipulate non-traditional animal models to ask genetic questions in parallel to naturalistic field approaches. It is our hope with this Commentary to show that experiments can be expanded or modified to cast behavior in a more naturalistic light and to inspire new questions, noting that the value of this approach is already being discussed in neuroscience (Krakauer et al., 2017; Yartsev, 2017; Mobbs et al., 2018; Gomez-Marin and Ghazanfar, 2019). Many features of natural environments are often unaccounted for or absent in laboratory experiments; thus, becoming reacquainted with our model systems in their native niche can help advance our understanding of their natural behaviors and contextualize observations made in the lab. Probing the system in its more natural state yields powerful insights that we may miss with traditional approaches that push a system to its limits (e.g. with artificial stimuli, genetic mutations and neuronal ablations).

Just as the pertinent conversation topics can vary greatly in personal relationships, scientists at different career stages and studying different animal systems can each discover unique value in exploring the 10 topics (Table 1) presented in this Commentary. For example, researchers may already be very familiar with the genetics and anatomy of their animal but might not yet have considered the effect of a natural diet (Q2). Researchers looking to expand to a sister species or a new organism due to its fascinating behavior may want to consider the requirements for housing (Q1), mating (Q7) and genetic mapping (Q4) for their new study system. Neuroscientists investigating neural computation during complex tasks could consider the effects of circadian rhythms on behavioral performance (Q3), incorporate the range of sensory inputs their model organism perceives (Q6) and examine biomechanical limits imposed on action selection (Q5).

**Table 1.**
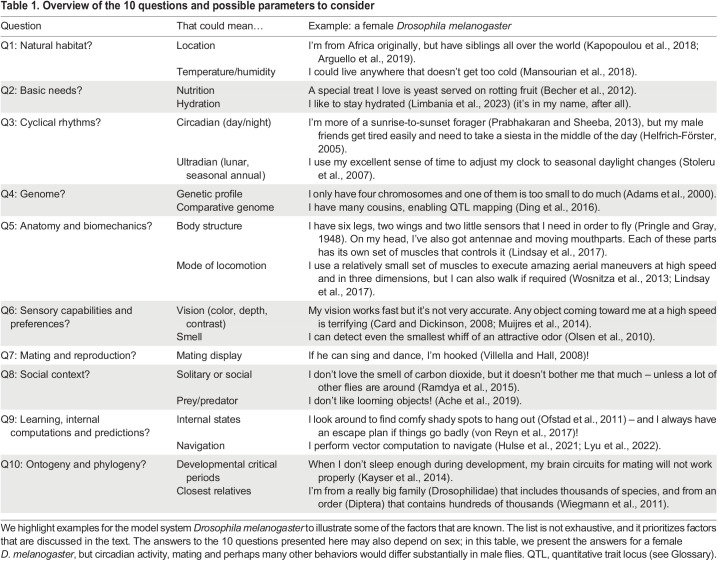
Overview of the 10 questions and possible parameters to consider

Our experiments are a continuous conversation with our animals. Here, we provide 10 questions (Table 1), cut down from the *New York Times*'s 36, to ask about your animal in the hope that it will provide an illuminating context in which to study the mechanisms underlying its behavior. Answering these questions will require bridging the gap between controlled laboratory conditions and natural habitats. We hope that these questions ignite a spark that makes you fall in love (again) with your organism. Staring into its eyes for 4 minutes is optional…if it even has eyes.

## The 10 questions

### Q1: Where are you from? Determining the natural habitat

Two approaches can bring out the best in your animal: bringing nature into the lab, or taking the lab into nature. The former is not trivial: many species are difficult to raise, keep or breed in the laboratory. Where is your animal going to live and with whom? Ideally, you need to raise numerous animals for experiments, but not all animals enjoy company. Octopuses and spiders will harm or even eat each other, so separate housing is often necessary, which means separate feedings and separate housing maintenance, for example (Zschokke and Herberstein, 2005). Conversely, social animals such as parrots are better housed in groups (Aydinonat et al., 2014). Studying an animal in the wrong experimental environment could result in artificial stress that may muddle our interpretation of their behavior (Kikusui et al., 2006).

Bringing salient aspects of the natural environment to the lab reveals new behaviors and neural functions (Dickinson et al., 2000; Liao et al., 2003; Lewarch and Hoekstra, 2018; Applegate and Aronov, 2022). For example, simply providing the roundworm *Caenorhabditis elegans* with a textured substrate allows it to showcase nictation behavior, a standing posture useful for hitching a ride on an insect carrier (Lee et al., 2012). Other worm behaviors, such as jumping, have been observed for the first time on substrates mimicking their natural compost-like environment (Guisnet et al., 2021). As for newly uncovered neural functions, recording from the hippocampus of bats flying freely in large spaces has revealed a new multiscale place-coding mechanism, one that is likely to be operational during large-scale natural migrations (Yartsev et al., 2011; Eliav et al., 2021). Similarly, flight behavior changes the directional sensitivity of neurons in the butterfly central complex (see Glossary) compared with those of animals on the ground (Beetz et al., 2022). Even in quiescent animals, naturalistic and artificial stimuli can affect neurons in very different ways. For example, auditory neurons encode natural sounds more efficiently than laboratory-created pure tones and extract more information from them (Rieke et al., 1995). Similarly, presenting retinal neurons with natural scenes, which contain spatial and temporal correlations that are not present in artificial stimuli, has revealed efficient coding mechanisms that reduce these redundancies (Pitkow and Meister, 2012).

Even more radical than bringing the field to the lab is to study animals in the habitats where they have evolved (Benítez et al., 2022). Even for popular laboratory organisms such as *C. elegans* or *Drosophila*, recent studies have focused on their natural ecology to contextualize our understanding of the behaviors we observe in the laboratory, and also provide background for sensory optimizations or adaptations that have evolved in these environments (Schulenburg and Félix, 2017; Mansourian et al., 2018; Leitch et al., 2021). Another option is bringing canonical laboratory animals into field enclosures or semi-natural lab environments (Graham, 2021; Zipple et al., 2023). A bridge between the mechanistic access of these species and their natural habitat, these experiments have already provided insights into the social structures and spatial distribution of mice (Vogt et al., 2023 preprint). When animals are left to their own devices, especially in large places, they can surprise us with behaviors that reveal their impact at the ecological level (Savoca et al., 2021).

### Q2: What's your favorite dish? Fulfilling basic needs

Animal behavior can vary wildly with nutrition: female black widow spiders often consume their mates following copulation, but supplementing their diet so they are not hungry makes them less likely to do this (Andrade, 1998). In most cases, laboratory chow is monotonous – nutritious but not exciting. We provide nematodes with *E. coli* they would not naturally encounter (Zečić et al., 2019) and mice with ready-made pellets rather than live insects (Galvin et al., 2021). Such simplifications come at a price: variables such as feeding rate, locomotion speed (Shtonda and Avery, 2006; Bonnard et al., 2022), predation (Akduman et al., 2020) in certain nematodes, egg-laying behavior in *Drosophila* (Becher et al., 2012) and impulsive choice behaviors in rats (Steele et al., 2017) can vary depending on diet. When we give our animals the task of choosing between multiple foods, their responses depend on several variables that may be easy to miss. For example, *Drosophila* seek out food based on their nutritional needs, with adult females preferring protein-rich yeast over sucrose to support resource-intensive egg production (Corrales-Carvajal et al., 2016). *Caenorhabditis elegans* chooses familiar over unfamiliar foods, presumably to avoid pathogens (Song et al., 2013), and prefers bacteria with smaller cell sizes that are easier to eat (Avery and Shtonda, 2003; Shtonda and Avery, 2006).

Diet also strongly influences the gut microbiome. Gut microbes can influence food intake (Jia et al., 2021; Ousey et al., 2023), behavior (Jia et al., 2021; Ousey et al., 2023) and health (Fontana and Partridge, 2015): in rats, for example, gut microbiota may enhance survival in human-dominated environments, with city-dwelling common black rats containing microflora different from those in non-commensal forest-dwelling species (Varudkar and Ramakrishnan, 2018). It is difficult to causally link microbiome composition to behavior, but there has been some success in invertebrate models (Petersen and Osvatic, 2018). In *C. elegans*, tyrosine produced by intestinal bacteria directly modulates aversion to olfactory cues (O'Donnell et al., 2020).

Considering diet as a factor in behavioral experiments opens up new avenues of investigation, for example, by unlocking new behaviors such as hunting in mice (Galvin et al., 2021), by allowing investigation of mechanisms of non-neuronal modulation of behavior via the gut–brain axis (O'Donnell et al., 2020), or by allowing manipulation of motivational state or energy balance to gain insight into organismal priorities (Song et al., 2013; Corrales-Carvajal et al., 2016).

### Q3: Are you an early bird or a night owl? Following cyclical rhythms

The circadian clock is an essential consideration when studying and interpreting animal behavior in the lab and in the field (Valentinuzzi et al., 2004). Many laboratory organisms live with artificial 24 h light/dark cycles, conveniently fixed to accommodate researchers' hours. There is no such luxury in the field: researchers must follow the daylight cycle and changing seasons (Prabhakaran and Sheeba, 2013) to capture activity peaks.

Although almost all animals have a circadian rhythm, there are exceptions: animals living in the lightless deep sea, some arctic animals that experience 24 h daylight in summer and constant darkness in winter (Bloch et al., 2013), and animals that bury deep underground or inside substrates (Beale et al., 2016). In the terrestrial nematode *C. elegans*, many genes regulating circadian rhythm in related species are repurposed to regulate developmental timing (Jeon et al., 1999). Longer cycles, such as lunar and annual rhythms, are also important for many animals. The waxing and waning of the moon alters nocturnal light, but also affects the tides, and many aquatic organisms link their reproductive behaviors to this cycle (Andreatta and Tessmar-Raible, 2020). Along the Great Barrier Reef, corals synchronize their spawning to the full moon during the summer, causing a mass eruption of eggs and sperm (Babcock et al., 1986). For animals in non-equatorial latitudes, anticipating the changing seasons (and hence food supply) has led to the evolution of a variety of seasonal behaviors, such as migration and hibernation. Asking about the relevant cyclicality helps us understand the behavioral patterns and variability in our animals and should crucially guide how we study them. Manipulating such cyclicality, for example, by using artificial light cycles to simulate 20 h days or temperature cues to signal seasonal changes, opens up new avenues of behavioral research.

### Q4: What genes are you wearing? Interrogating the genome

The rapid development of sequencing technologies and assembly algorithms has dramatically accelerated the generation of genomes, transcriptomes and single-cell atlases (see Glossary) that are invaluable resources for behavioral research. Although behavior is several steps removed from genetics, specific genes can strongly influence behavior. For example, many animal behaviors are influenced by olfaction, and the diversification of olfactory receptors and their underlying circuits alter not only how animals understand their environment but also how they identify conspecifics (Seeholzer et al., 2018; Auer et al., 2020).

With a genome in hand, the evolution of behaviors in the wild can be explained through selective pressure at specific loci. The Mexican tetra *Astyanax mexicanus* is an excellent example of this. Populations of subterranean cavefish have independently evolved several times from surface populations. These morphs are often blind, albino and exhibit behaviors such as loss of sleep (Yoshizawa et al., 2015) and schooling (Kowalko et al., 2013), reduced aggression (Elipot et al., 2013) and increased lateral line sensitivity (Yoshizawa et al., 2010; Lunsford et al., 2022). Although the loss of functional eyes has evolved independently at different genomic loci in different cave populations, disrupted circadian rhythms have evolved through novel deletions in the same gene: *oca2*, which confers pleiotropic traits (see Glossary) such as albinism and sleep loss (O'Gorman et al., 2021). Without an understanding of the genome, many of the tetra's behaviors would remain mysterious. However, advances in sequencing technology have made this, and future studies, far more feasible. This knowledge of the genome, combined with gene-editing technologies such as CRISPR, provides us with invaluable resources to interrogate the genetics of any organism of our choosing.

### Q5: What physical attributes are you attracted to? Examining anatomy and biomechanics

Exceptional anatomy often reveals exceptional behavior. If we understand the interconnections of muscles, bones and neurons, for example, we can predict how parts of the body can move (Tytell et al., 2011). For example, the ascending process of a sling jaw wrasse's mouth (*Epibulus insidiator*) alerts us to extreme jaw protrusion that is useful in piscivory on coral reefs (Westneat, 1991; Burgess et al., 2011), as much as the synaptic connection between two neurons signifies a working relationship. And although anatomy is partly inherited, it is also at the mercy of the developmental environment. The nematode *Pristionchus pacificus* develops into two distinct adult morphs depending on food availability at the larval stage: a predatory toothed morph that feeds on other nematodes, and a solitary morph that feeds on bacteria (Werner et al., 2017). In this way, anatomy can be sculpted by life history.

Moving animals have evolved within the physics of the real world. We cannot truly appreciate behavior without understanding the biomechanics of movement. For example, the efficiency of acceleration in swimming fishes is constrained by the hydrodynamics of optimal vortex formation across many fish species regardless of body shape (Akanyeti et al., 2017). Likewise, camels, which are adapted to a flat desert terrain, predominantly use a pacing gait that is unstable in rugged environments and not employed by alpacas, which live in mountainous terrain (Dagg, 1974; Pfau et al., 2011). The physics of the available substrates also limit what behaviors can be observed. For example, in the quasi-2D environment of most experimental setups, mice do not have the opportunity to burrow, whereas in their natural habitats, many species show remarkable innovations in burrowing – their enriched environment unlocking previously hidden biomechanics (Hu and Hoekstra, 2017).

The mechanical properties of the body and how it interacts with the environment when it moves dictate many of the details of behavioral performance and its costs (Hunt et al., 2021; Jimenez et al., 2023). In some cases, animal cadavers can move passively: a dead fish towed in unsteady flows undulates similarly to a live fish, and generates thrust using an energy-recapture mechanism analogous to that exploited in surfing (Beal et al., 2006). The body and its intrinsic elasticity can still perform a surprising range of movements without input from the brain, as in animals that have undergone pithing (see Glossary) or are even dead (Lundberg and Phillips, 1973); yet, the brains of animals can do nothing without the body.

### Q6: What are you listening to? Understanding sensory capabilities and preferences

An experiment on an animal whose senses are overwhelmed will end poorly (Cherry, 1953; Moray, 1959), and our own sensory biases may trick us into ignoring cues our animals can sense but we cannot (Caves et al., 2019; Yong, 2022). This concept was termed ‘umwelten’ by Jakob von Uexküll (Caves et al., 2019), who argued that differences in sensory systems mean that each animal species experiences essentially a different world. Hearing is a classic example: humans can only hear up to 20 kHz, whereas rats communicate (Anderson, 1954) affection in response to both intra-species touch and human tickling (Ishiyama and Brecht, 2016; Hinchcliffe et al., 2020) at 50 kHz, completely outside our auditory detection. We generally assume that animals can sense the same things we can, and then discover that their senses often go beyond ours [e.g. UV vision (Losey et al., 1999; Honkavaara et al., 2002), the polarization compass in insects (Dacke et al., 2003) or the lateral line in fishes (Coombs and Montgomery, 1999)]. Animals dazzle us with perceptive abilities that challenge our imagination; from echolocation (Surlykke and Moss, 2000) to electroreception (Bullock et al., 2005), our world abounds with physical cues to which only certain animals are privy.

Many animals face the challenge of loud environments, e.g. near waterfalls (Arch et al., 2008), and nervous systems have evolved mechanisms for gain modulation (see Glossary) to allow animals to focus on specific sensory inputs (Andersen and Mountcastle, 1983; Ghazanfar and Logothetis, 2003; Williamson et al., 2016). One solution to noisy environments is used by Emperor penguins, which solve the challenge of finding their chicks amongst the loud squawks of the colony by producing unique beats from their two-part sound organ, to which only their chicks respond (Aubin et al., 2000).

Sensory systems function under many different circumstances, and the impact of unwanted sensory input cannot be ignored. When designing experiments, the stimulus you introduce may not be the only stimulus that steers the behavior of your animal. For example, immobilizing a larval zebrafish, as typically required for electrophysiology experiments, produces sensations that make it less likely to perform C-start escape behaviors (see Glossary; Liao and Fetcho, 2008; Mu et al., 2019; Bhandiwad et al., 2022). Tethering a fly to a pin makes it possible to study its visual system in detail (Reiser and Dickinson, 2010) but removes the proprioceptive inputs that occur in free flight, thus changing the fly's behavior (Rimniceanu et al., 2023). It might seem obvious that we should consider both wanted and unwanted stimuli, but we may yet be surprised as new senses or expanded sensory ranges are discovered.

### Q7: What kind of partner are you looking for? Considering mating and mode of reproduction

In the Darwinian sense, all biology revolves around an animal's ability to reproduce, and nature is filled with evolutionary solutions to this challenge: some animals have complex elaborations of their mating rituals and displays [e.g. the Vogelkop bird-of-paradise courtship dance (Scholes and Laman, 2018), the immense geometric nest of the white-spotted pufferfish (Mizuuchi et al., 2018)], others can switch their reproductive mode or sex roles based on environmental cues [e.g. clownfish and other coral reef fishes (Fricke, 1983; Grafe and Linsenmair, 1989), common reed frog (Fricke, 1983; Grafe and Linsenmair, 1989), African butterfly (Brakefield et al., 2009)], some engage in reproductive division of labor (e.g. eusocial bees, ants, naked mole rats; Gadagkar, 1990), yet others are born with multiple sets of reproductive organs (e.g. slugs; Leonard et al., 2002), and some just do away with the partner search process altogether [e.g. asexual whiptail lizards (Lutes et al., 2010), sharks that switch from sexual to parthenogenetic reproduction (Dudgeon et al., 2017)].

For sexually reproducing animals, understanding their mating drive goes a long way to unraveling behavioral complexities. Mature *C. elegans* males prioritize mate search over feeding, but change their priorities if they have recently mated (reviewed in Barrios, 2014). For cooperatively breeding white-browed sparrow weavers, the dominant male performs distinct behaviors to protect and maintain mating access (Walker et al., 2016), such as sentineling against predation and being aggressive towards intruding males. Similarly, in male cichlid fishes, the presence of fewer egg-spots on the anal fin causes increased attacks and aggression from other males (Theis et al., 2012). After successful mating, altruistic dispersal behavior of male animals in some contexts is thought to theoretically conserve limiting resources for the expanding population (Starrfelt and Kokko, 2012).

Understanding a species' reproduction is also necessary for us to culture and study our animals in the laboratory. Even for classic model organisms that are typically easy to propagate, knowing their preferred mode for reproducing can improve how effectively they breed in captivity or how we select experimental controls, and can enable questions about parental care, population dynamics and ecology. Rearing wild animals in the lab can be challenging if the proper environmental cues are not present (Schulte-Hostedde and Mastromonaco, 2015). In some cases, replicating just the right conditions can encourage animals to mate: a gentle sprinkle of water on the surface of a fish tank, simulating rain, unlocks a suite of reproductive behaviors in electric fish (Wong and Hopkins, 2007). Conversely, the lab can create new environmental pressures that can alter mating behavior; for example, the photoperiod of their native latitude and seasonality alter the timing of sexual maturity in deer mice, deer and gray wolves (Schulte-Hostedde and Mastromonaco, 2015). As we expand the list of organisms we study in the lab or observe in the wild, we should not shy away from animals with very specific needs. The diversification of sexual behavior provides us with a powerful tool to understand the evolution of behavior, but this is only feasible if we sample across species using a comparative approach.

### Q8: Who are your friends and enemies? Scrutinizing the social and predator–prey contexts

An animal's conspecifics are competitors for food and mates, but animal collectives that show task distribution and specializations can develop entirely different behaviors from those of a solitary animal (Marras and Domenici, 2013). For many social animals, socialization itself is a reward, and experiencing social contact with conspecifics is often critical to functional behaviors as an adult (Nardou et al., 2019; Wang et al., 2022). In contrast, some animals would rather avoid or even eat same-sex conspecifics, as they represent competitors for food and mates (Vijendravarma, 2023). Sophisticated behaviors can also emerge from individuals operating on relatively simple nearest-neighbor algorithms without an inherent social structure (Bialek et al., 2012; Ding et al., 2019): at high density, locust swarms start migrating due to each individual avoiding being eaten by a conspecific (Guttal et al., 2012), and some fireflies synchronize their pulses with the flashes from another (Sarfati et al., 2023).

The presence of parasites in or on an animal's body can drastically influence its behavior, as illustrated by the classic example of rats infected with the protozoan *Toxoplasmosis*, which become attracted to the odor of cat urine (House et al., 2011), and ants infected with the fungi *Ophiocordyceps unilateralis*, which clamp their mandibles onto vegetation and remain there until death, making them easy targets for predators and thus completing the life cycle of the fungus (Andersen et al., 2009). This parasitic manipulation can also affect social behavior: in ants, parasitic nematodes change the colony's division of labor to allow the parasite to better spread from individual to individual (Li et al., 2023). Further, some parasites can change the behavior of third parties; this can occur in systems involving hyperparasites, which parasitize parasites (Kaplan, 2012). Investigation of these increasingly complex interactions is in its infancy, and is likely to impact our view of natural communities in the coming decade, including effects on social interactions and predator avoidance.

Unless your study animal is an apex predator, it will always be on the lookout for things that want to eat it. Studying the implications of realistic predator–prey interactions in naturally occurring populations is very challenging, although not impossible (Reznick, 1982). In the laboratory, tractable natural predator–prey systems reveal surprising new behaviors. For example, the nematode *P. pacificus* preys on *C. elegans* larvae, but both species also consume bacteria from their environment. By studying this intraguild predation under the microscope, we now know that *P. pacificus* flexibly switches between two biting modes against *C. elegans*: one for killing them as prey and the other as a deterrent to defend their bacterial food (Quach and Chalasani, 2022).

How much realism is required when investigating predator–prey systems and social interactions? In the lab, artificial looming predator stimuli reliably elicit escape responses in fruit flies and mice (Card and Dickinson, 2008; Yilmaz and Meister, 2013), suggesting that escape behavior is robust and does not require fully realistic stimuli. This makes sense, as an overly cautious response to danger is often less detrimental than a false negative. In contrast, positive socialization requires more care to evoke; for example, rat mothers respond only to very specific pup vocalizations but pure tones will not evoke maternal care behavior (Valtcheva et al., 2023). Modern tools such as tracking technologies and robots mimicking conspecifics or predators (Choi and Kim, 2010) have much to offer for disentangling the complexities inherent in social interactions and predator–prey dynamics.

### Q9: What are you thinking about right now? Appreciating an animal's learning, internal computations and predictions

Acknowledging what your animal experiences and expects is essential for interpreting behavior. Neural activity in an organism can reflect pervasive internal states (such as fear or hunger; Flavell et al., 2022), represent behaviorally relevant variables [spatial surroundings (Kay et al., 2020), attention (Fiebelkorn and Kastner, 2019)], correspond to spontaneous activity even in the absence of an obvious stimulus or task (Kato et al., 2015; Stringer et al., 2019; Avitan et al., 2021; Hallinen et al., 2021; Avitan and Stringer, 2022), or, as often happens in biology, be a messy mixture of all of the above. Many animals develop specific preferences based on their experience: rodents associate certain handlers with specific procedures (Sorge et al., 2014; Sensini et al., 2020), mosquitoes react to scents of the experimenters (De Obaldia et al., 2022), even worms learn to recognize the temperature and food they were raised on and will prefer those conditions later in life (Hedgecock and Russell, 1975; Kimura et al., 2004; Song et al., 2013). On a slightly faster time scale, there are many examples of behavioral changes that correspond to changes in neural activity patterns: hungry larval zebrafish switch to a hunting-like behavior state as they alter the neural representation of prey-like stimuli in the tectum (Filosa et al., 2016); fear-conditioned rats show active place avoidance while preferentially reactivating place cells corresponding to the shocked zone (Wu et al., 2017); arousal modulates resting versus active brain states in a wide variety of organisms and is correlated with brain-wide expression of movement-active neurons (Ahrens and Engert, 2015; Kato et al., 2015; Kropff et al., 2015; Nguyen et al., 2016; Venkatachalam et al., 2016; Musall et al., 2019; Hallinen et al., 2021); mice switch between exploration and exploitation in a pattern that can be predicted by changes in the population code in the amygdala (Gründemann et al., 2019).

However, sometimes animals do not have days, hours or even minutes, but instead need to make split-second decisions. It is useful to know at which time scales our animals can integrate current sensory information and predict possible future scenarios, and how these representations interact with each other during the complexity of natural behaviors we observe. In rodents, hippocampal population activity shows internal representations of both current and hypothetical spatial locations at fast time scales during locomotion (Skaggs et al., 1996; Wang et al., 2015; Kay et al., 2020; Wang et al., 2020; Joshi et al., 2023), which is likely to enable constant sensory processing and future action selection (Tolman et al., 1946; Comrie et al., 2022). High-density neural recordings with imaging techniques and flexible probes are developing at a rapid pace (Ahrens and Engert, 2015; Jun et al., 2017; Voleti et al., 2019; Markicevic et al., 2021) to reveal how pervasive internal states interact with ongoing neural activity. These techniques can increasingly be implemented in your favorite model organism to investigate how neural computations across time scales may contribute to ongoing actions.

### Q10: Can you tell me about your family? Accounting for ontogeny and phylogeny

Childhood experiences can shed light on adult behavior, as some behavioral patterns are only learned early in childhood. For example, zebra finches must be exposed to song tutors during a sensitive period, ∼25–65 days post-hatch, or they will fail to learn the parent's song (Gobes et al., 2019); in mice, social reward (see Glossary) must be learned within a critical period during juvenile development (Nardou et al., 2019). Neglecting an animal's native ethological context when raising juveniles outside of their natural environment can inadvertently alter behavior in adults.

In addition to their own life history, the family history can also tell you a lot about your animal. For example, most nematodes have complex life cycles where different developmental stages are either parasitic or free-living. Although the popular model nematode *C. elegans* is free-living (Bryant et al., 2022), its sister species *Caenorhabditis inopinata* inhabits figs (*Ficus septica*; Kanzaki et al., 2018). During a special developmental stage (dauer), the nematodes nictate, vertically standing on their tails to improve their chances of attaching to a wasp as it lands on a fruit. The wasp carries the animals to new fruit, ensuring *C. inopinata*'s dispersal. *Caenorhabditis elegans* appears to be more of a generalist in the wild, but the behavior of its sister species explains how dauer behaviors evolved and why the free-living *C. elegans* still shows nictation behavior.

In some cases, natural variation in the genes of related species can be used to infer their role in behavior. If introgression (see Glossary) is possible in the laboratory, behavioral variation may be linked to genetic variation through quantitative trait locus (QTL) analysis. Two closely related species of *Peromyscus* mice construct burrows, but *P. maniculatus* only builds short burrows as an adult, whereas *P. polionotus* has precocious building as a juvenile and produces longer, more elaborate burrows as an adult (Weber et al., 2013; Metz et al., 2017). As these species have mapped single nucleotide polymorphisms and can be introgressed, QTL analysis on burrow complexity can be used to map the behavior to specific genomic loci. Further work is needed to resolve the causative alleles in these loci, but this approach can be fruitful for other wild species with divergent phenotypes and natural hybrids that exist between two species.

## Conclusion

Now is the time to embrace a deeper understanding of animal behavior: armed with powerful new technologies unheard of even a decade ago, researchers can explore new behaviors in both model systems and non-traditional species. Experiments inspired by fieldwork can now be reconstructed in the laboratory with higher fidelity than ever before. As more species become amenable to quantitative approaches and new analyses, collaborations between scientists in the field and the lab promise to bring new models to neuroethology. For traditional models such as *C. elegans* and *D. melanogaster*, burgeoning knowledge of their natural history and ecology, as well as the investigation of sister species or populations from different niches, has provided important insights into the evolution of behavior (Félix and Duveau, 2012; Frézal and Félix, 2015; Auer et al., 2021).

In this Commentary, we have aimed to provide an overview of the many factors to consider when studying animal behavior. We have provided examples of experimental approaches that have bridged some gaps where our understanding may be lacking, and shared evidence for other essential gaps that are yet to be filled. Our goal is that this 10-question framework will serve as an initial guide for studying the mechanisms underlying animal behavior. It may also provide an evolutionary or ecological context to better understand a species, to unveil alternative states of a well-studied behavior or even unlock entirely new behaviors. Many relationships, whether just beginning or having celebrated many anniversaries, can benefit from genuine curiosity and a deeper understanding of the other party; the same is true for your organism of study. Whatever you find, we hope that you enjoy the conversation.

## ECR Spotlight

This article has an associated ECR Spotlight interview with Abhilasha Joshi.
